# Magnetization of Wiegand Wires with Varying Diameters and Analysis of Their Magnetic Structure via Hysteresis Loops

**DOI:** 10.3390/ma16093559

**Published:** 2023-05-06

**Authors:** Liang Jiang, Chao Yang, Zenglu Song, Yasushi Takemura

**Affiliations:** 1 Department of Electrical and Computer Engineering, Yokohama National University, Yokohama 240-8501, Japan; 2 School of Electrical Engineering, Nanjing Vocational University of Industry Technology, Nanjing 210023, China

**Keywords:** Wiegand wire, hysteresis loop, magnetization process, large Barkhausen jump, magnetic structure

## Abstract

Wiegand wires are unique ferromagnetic materials that display rapid magnetization reversal and a large Barkhausen jump under an applied field. This stable reversal can be used to induce a periodic pulse voltage in a pickup coil wrapped around the Wiegand wire. To unlock the full potential of Wiegand wires for magnetic sensors and devices, the magnetic structure and magnetization state of the Wiegand wire must be fully elucidated. In this study, hysteresis loops were used to reveal the magnetic structure of Wiegand wires. Wiegand wires of different diameters magnetized under different applied magnetic field strengths were analyzed in detail. Our results show that Wiegand wires 0.06 mm in diameter are composed solely of a hard magnetic core. Wiegand wires above 0.10 mm in diameter have a hard magnetic core, a middle layer, and a soft layer that decreases in thickness but increases in coercivity as the wire diameter decreases. Then, theoretical models were built to predict the magnetic structure of Wiegand wires under an applied field for the first time. The magnetization process of Wiegand wires with different diameters under different applied magnetic fields was also analyzed.

## 1. Introduction

The Wiegand effect, which involves rapid magnetization reversal in magnetic wires accompanied by a large Barkhausen jump, was first realized technically by John Wiegand [1,2]. The Vicalloy Fe_0.4_Co_0.5_V_0.1_ is the optimum ferromagnetic material for generating the Wiegand effect [3,4,5]. Consequently, Fe_0.4_Co_0.5_V_0.1_ wires are synonymously called Wiegand wires [6]. Fe_0.4_Co_0.5_V_0.1_ exhibits bistable characteristics and is prepared by cyclically applying torsional strain and longitudinal strain to the ferromagnetic material [7]. The outer and inner layers of Wiegand wires exhibit magnetically soft and hard properties with low and high coercivities, respectively [8]. The magnetically soft layer first undergoes rapid magnetization reversal followed by a large Barkhausen jump under an alternating applied magnetic field [9,10]. A Wiegand sensor can be created by winding a pickup coil around the Wiegand wire. A stable pulse voltage can be generated in the pickup coil when an alternating magnetic field is applied to the Wiegand sensor [11]. The generated pulse voltage is independent of the frequency of the applied magnetic field, making Wiegand sensors advantageous over conventional sensors [12].

Many applications for Wiegand wires have been developed. Wiegand wires are widely used for the battery-less operation of rotary encoders [13]. They can also be utilized as a power source for microelectronic devices [14]. Wiegand wires have also been used for energy collection and wireless energy transmission [15,16]. To improve the operational performance of Wiegand wires and expand their range of applications, it is particularly important to predict the magnetic structure of Wiegand wires under different applied magnetic fields. To date, this has rarely been investigated, and there are large gaps in knowledge regarding the effect of magnetization in Wiegand wires. A magnetic field camera based on the Faraday effect was used to directly observe magnetization reversal in Wiegand wires [17]. The magneto-optical Kerr effect was used to measure the magnetization on the surface of a Wiegand wire [18]. The magnetization of the various layers in the Wiegand wire can be identified using first-order reversal curve (FORC) diagrams [19,20,21]. Recently, the magnetization reversal behavior of single and several sets of amorphous microwires under applied magnetic fields can be obtained by using the FORC method. The FORC model can well analyze the coercive and interaction field distributions of single and several sets of amorphous microwires, which cannot be obtained by hysteresis loops [22]. However, the abovementioned analyses were qualitative, and the magnetic structure and magnetization process under different applied magnetic fields were not explained in detail.

This study aims to clarify the magnetic structure, determine the radial thickness of the magnetized and reverse magnetized layers, and explain the magnetization process in Wiegand wires of varying diameters under different applied magnetic fields along the wire length direction (easy axis) in detail by analyzing their magnetic hysteresis loops [21]. The major and minor hysteresis loops of Wiegand wires of varying diameters were measured using a vibrating sample magnetometer (VSM). The thin wire has a large coercivity on average, and the thick wire has a small coercivity on average. Based on a three-layer magnetic structure [20], the thicknesses of the soft and middle layers were determined by calculating the ratio of the magnetization change originating from the large Barkhausen jump to the saturation magnetization. Therefore, a simple magnetic structure delineating the soft, middle, and hard layers of the Wiegand wires was proposed for the first time by theoretical calculations. Another novel contribution of this study is that the thicknesses of the magnetized regions with each direction under an applied magnetic field can be calculated for the first time. A theoretical model for analyzing the magnetization process in Wiegand wires under different applied magnetic fields was then developed. Thus, the magnetization state and magnetization reversal state in Wiegand wires during the entire magnetization process can be accurately calculated at each point on the minor hysteresis loop.

## 2. Materials and Methods

**Figure 1:**
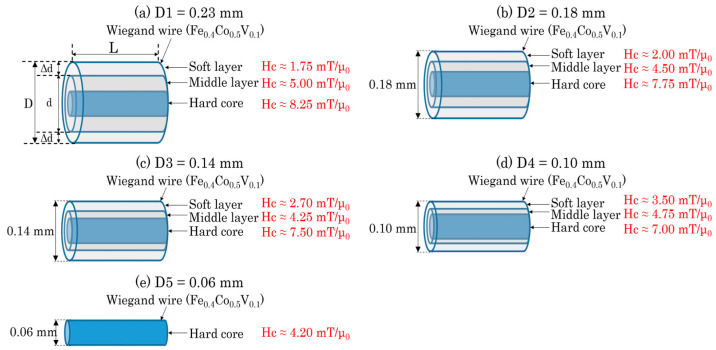
Magnetic structure of Wiegand wires with varying diameters [20].

Wiegand wires 13 mm in length and 0.23 mm in diameter (SWFE, Co. Ltd., Meishan, China) were prepared in this study. To analyze the magnetic structure of the Wiegand wire in the radial direction, the outer surfaces of four wires were etched with FeCl_3_ solution to varying degrees. Consequently, five Wiegand wires labeled D1, D2, D3, D4, and D5, with diameters of 0.23, 0.18, 0.14, 0.10, and 0.06 mm, respectively, were prepared. Their magnetic structures were analyzed in detail in a previous study [20]. The 0.06-mm-diameter Wiegand wire (D5) has a single and uniform magnetic structure with a coercivity of approximately 4.20 mT. The magnetic structure of the other Wiegand wires comprises three magnetic layers: the soft, middle, and hard layers. The magnetic structures of the five Wiegand wires are depicted in Figure 1. The coercivity of each layer of the Wiegand wires was determined [20]. The coercivity of the soft and hard layers of D1 was 1.75 mT and 8.25 mT, respectively, which is consistent with previous research [23]. Figure 1 also shows that with a decrease in the diameter of the wire (D1 to D2 to D3 to D4), the coercivity of the soft layer gradually increases, while that of the hard layer gradually decreases.

Hysteresis loops are commonly used to evaluate the magnetic characteristics of magnetic materials. Hysteresis loops provide essential information regarding induced and remnant magnetizations. For instance, the height of the large Barkhausen jump and the magnetization of the Wiegand wires used in this study can be obtained from the magnetic hysteresis loop. Therefore, the thicknesses of the magnetized regions in each direction of the Wiegand wires can be evaluated. In this study, the major and minor hysteresis loops of the Wiegand wires were measured using a VSM (Model 8600 series, Lake Shore Cryotronics, Westerville, OH, USA) at room temperature [20]. All measurements were completed in the previous paper. This study is a further analysis based on the measurement results and conclusions obtained in previous papers.

## 3. Results

### 3.1. Major Hysteresis Loops

**Figure 2:**
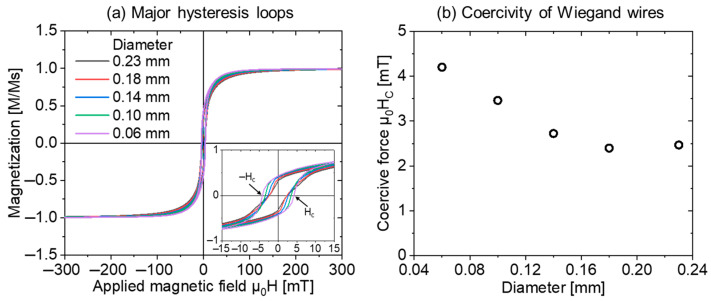
(**a**) Major hysteresis loops and (**b**) coercivity of Wiegand wires with different diameters [20].

The normalized major hysteresis loops of the Wiegand wires (D1, D2, D3, D4, D5) are shown in Figure 2a, with the origin magnified and embedded. The width of the hysteresis loop with the horizontal axis at zero magnetization is significant when the diameter of the Wiegand wire is small. That is, the smaller the diameter of the Wiegand wire, the greater the coercivity. The variation trend of the coercivity of the Wiegand wires with different diameters is shown in Figure 2b. This clearly demonstrates that the coercivity decreases as the diameter of the wire increases.

### 3.2. Minor Hysteresis Loops

**Figure 3:**
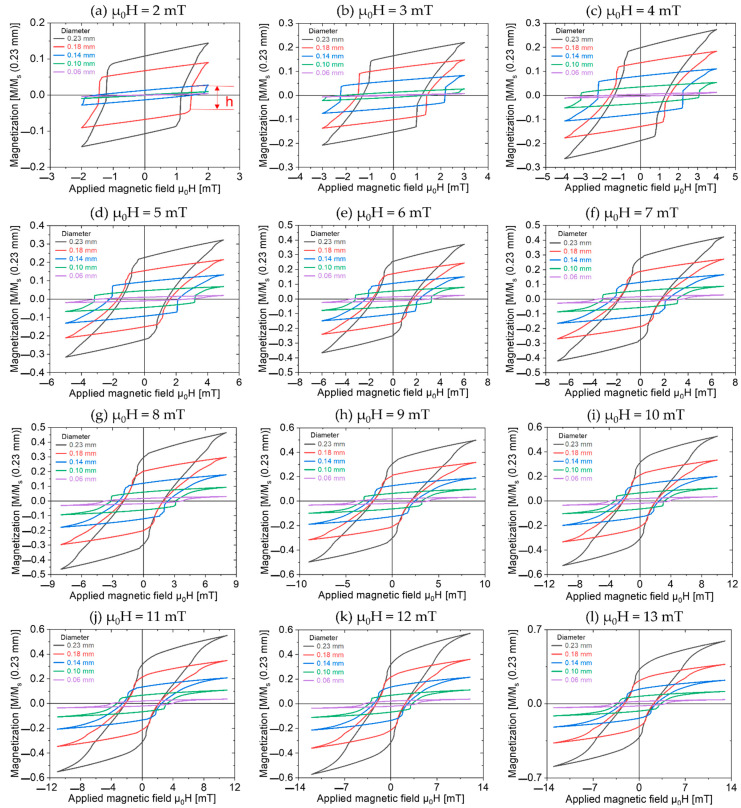

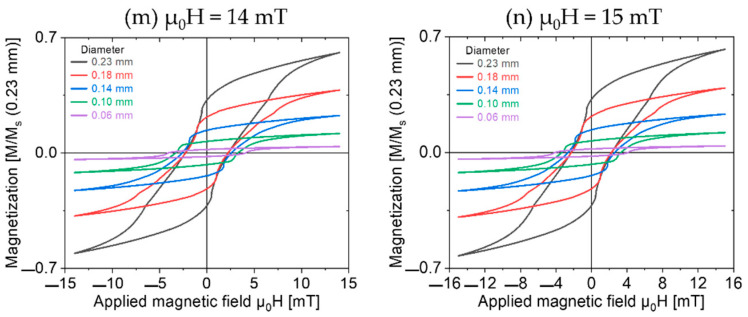
Minor hysteresis loops of Wiegand wires normalized by 0.23 mm diameter Wiegand wire.

The minor loops of the Wiegand wires were measured, as shown in Figure 3. An alternating magnetic field (μ_0_H) from 2 to 15 mT was applied with a step size of 1 mT. The Wiegand wires were normalized to the 0.23 mm diameter Wiegand wire, where h is the height of the large Barkhausen jump.

## 4. Discussion

### 4.1. The Simple Magnetic Structure of Wiegand Wires with Varying Diameters

**Figure 4:**
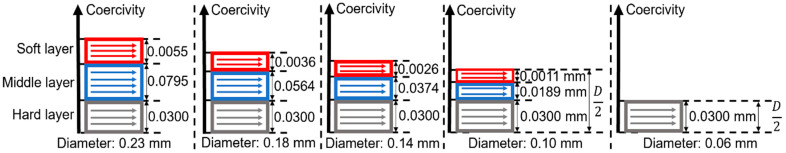
Schematic diagram of the simple magnetic structure of Wiegand wires with varying diameters.

From FORC analysis, it was established that the 0.06 mm diameter Wiegand wire is composed of a single hard core; therefore, the thickness of the hard layer can be set as 0.06 mm. The magnetic structure of the Wiegand wires with diameters above 0.06 mm consists of three layers: soft, middle, and hard [20]. A large Barkhausen jump is generated by the magnetization reversal in the soft layer [16]. The height of the large Barkhausen jump is proportional to the volume of the soft layer. Therefore, the thickness of the soft layer can be calculated using Equations (1) and (2):

Δd = (D − d)/2(1)

(2) $$\frac{(\pi d^{2}L)/4}{(\pi D^{2}L)/4}=\frac{1-\;h}{1}$$

where Δd is the thickness of the soft layer, d is the thickness of the middle and hard layers, D is the diameter of the Wiegand wire, and L is its length (see Figure 1a). The largest height of a large Barkhausen jump can be expressed by h. Thus, we can obtain the simple magnetic structures of the five Wiegand wires (see Figure 4). Because the magnetic structure of a Wiegand wire displays cylindrical symmetry, we can describe the semi-cross-sectional structure in the axial direction.

As shown in Figure 4, as the diameter of the Wiegand wire decreases, the thicknesses of the soft and middle layers decrease until only the hard core remains. To simplify the analysis and obtain the magnetic structure models in Figure 4, it was assumed that the thickness of the hard core of the Wiegand wire does not change during the etching process because it is located in the innermost part of the wire. The diameter of the Wiegand wire was etched from 0.23 mm to 0.18 mm, and the thickness of the outer layer was reduced by 0.025 mm, which was greater than 0.0055 mm (the thickness of the soft layer in the 0.23 mm diameter Wiegand wire). In this case, it can be considered that the soft layer was completely etched away, leaving only the middle layer and the hard core. However, it can be seen from Figure 3 that there are still large Barkhausen jumps in the minor hysteresis loops of the 0.18 mm diameter Wiegand wire. Even when the applied magnetic field was only 2 mT, a large Barkhausen jump was observed. This indicates that there is a soft layer that can generate a large Barkhausen jump in the 0.18 mm diameter Wiegand wire. The coercivity of the soft layer was less than or equal to 2 mT (which is consistent with the result in Figure 1b) [20]. This is because while the outer soft layer was etched away, the remaining middle layer formed a new soft layer on the surface, and a new middle layer was formed. After the soft layer was etched away, the thicknesses of the hard and soft layers in the Wiegand wire changed, and the interaction, magnetoelastic, and magnetostriction between them also changed; thus, the coercivity of the new soft layer, middle layer, and hard core also changed. This analysis holds true for the Wiegand wires etched from 0.18 mm to 0.14 mm and finally down to 0.10 mm.

### 4.2. Model for Calculating the Volume of Region of Magnetization

**Figure 5:**
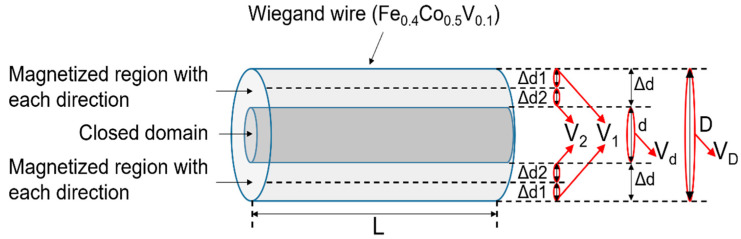
Magnetic structure of Wiegand wires with varying diameters.

The magnetic structures of the Wiegand wires with varying diameters are shown in Figure 5. Δd1 is the thickness of the region with reversed magnetization accompanied by a large Barkhausen jump. Δd2 is the thickness of the region whose magnetization was not reversed during the large Barkhausen jump. Δd = Δd1 + Δd2, as shown in Equation (3), where d denotes the thickness of the closed domain, and D and L are the diameter and length of the Wiegand wire, respectively. V_1_, V_2_, V_d_, and V_D_ represent the volumes of Δd1, Δd2, d, and D, respectively.

**Figure 6:**
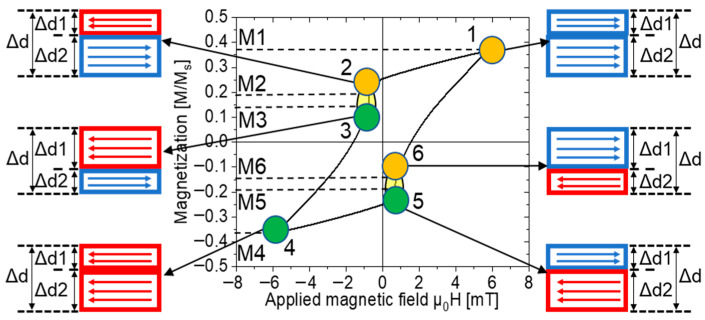
Magnetization process in an applied magnetic field.

The magnetization process under the applied magnetic field is shown in Figure 6. The numbers 1–6 are six points on the minor hysteresis loop. M1 and M4 are the maximum and minimum values, respectively, of magnetization under the applied field. M2 and M5 are the magnetization values before a large Barkhausen jump. M3 and M6 are the magnetization values after a large Barkhausen jump. M1 and M4, M2 and M5, and M3 and M6 were individually symmetric to the origin. The values of Δd, Δd1, and Δd2 in Figure 6 are identical to those in Figure 5. MX (X = 1, 2, 3, 4, 5, and 6) is the normalized magnetization value. When the Wiegand wire was fully magnetized, the values of M1 and M4 were 1 or −1. M1 and M4 are proportional to the volume of Δd. Thus, we can calculate the thickness of Δd using Equations (3) and (4).

Δd = (D − d)/2 = Δd1 + Δd2(3)

(4) $$\frac{V_{D}-{\;V}_{d}}{V_{D}}=\frac{\left|\mathrm{MX}\right|}{1}$$

In Figure 6, to simplify the model, it was assumed that there are only two magnetization directions: one to the left and the other to the right. M1 is positive, and the magnetization direction is to the right. M4 is negative, and its magnetization direction is to the left. In M2 and M3, the magnetization direction of Δd1 is to the left, and Δd2 is to the right. Thus, M2 and M3 were proportional to the volume (V_2_ − V_1_). In M5 and M6, the magnetization direction of Δd1 is to the right, and Δd2 is to the left. Thus, M5 and M6 were proportional to the volume (V_1_ − V_2_). Therefore, the thicknesses of Δd1 and Δd2 in M2 and M3 can be calculated using Equations (3) and (5), respectively. The thicknesses of Δd1 and Δd2 in M5 and M6 can be calculated using Equations (3) and (6), respectively.

(5) $$\frac{V_{2}-{\;V}_{1}}{V_{D}}=\frac{\mathrm{MX}}{1}$$

(6) $$\frac{V_{1}-{\;V}_{2}}{V_{D}}=\frac{\mathrm{MX}}{1}$$

In Equations (4)–(6), V_D_ and V_d_ can be calculated using the equations $(\pi D^{2}L)/4\;$ and $(\pi d^{2}L)/4$, respectively. V_1_ can be calculated using the equation $\pi (D/2{)}^{2}L\;-\;\pi (d/2+\Delta d2{)}^{2}L$, and V_2_ can be calculated using the equation $\pi (d/2+\Delta d2{)}^{2}L\;-\;\pi (d/2{)}^{2}L$.

Consequently, d can be calculated by Equation (7):

(7) $$d=\sqrt{1-\left|\mathrm{MX}\right|}$$

Δd2 in M2 or M3 can be calculated by Equation (8):

(8) $$\Delta d2=\left(\sqrt{\left[D^{2}\left(1+\mathrm{MX}\right)+d^{2}\right]/2}-\;d\right)/2$$

Δd2 in M5 or M6 can be calculated by Equation (9):

(9) $$\Delta d2=\left(\sqrt{\left[D^{2}\left(1-\mathrm{MX}\right)+d^{2}\right]/2}-\;d\right)/2$$

Using the above analysis, we can calculate the volume of the region with each direction of magnetization and the thicknesses of the reversed and unreversed volumes under an applied magnetic field. We used a semi-cross-sectional structure in the axial direction, as shown in Figure 4, to analyze the magnetization process and the complex magnetic structure of Wiegand wires with varying diameters. The applied magnetic field ranges from 2 mT to 15 mT. The calculated data for an applied magnetic field of 2 mT are shown in Table 1. As shown in Figure 3a, there was no large Barkhausen jump in the 0.10 mm diameter Wiegand wire when the applied magnetic field was 2 mT. The magnetization processes and complex magnetic structures of the Wiegand wires under different applied fields in this study are similar. Therefore, we selected certain typical magnetization processes and complex magnetic structures for the analysis.

### 4.3. Magnetization Process and Magnetic Structure of Wiegand Wires with Varying Diameters in an Applied Magnetic Field

**Figure 7:**
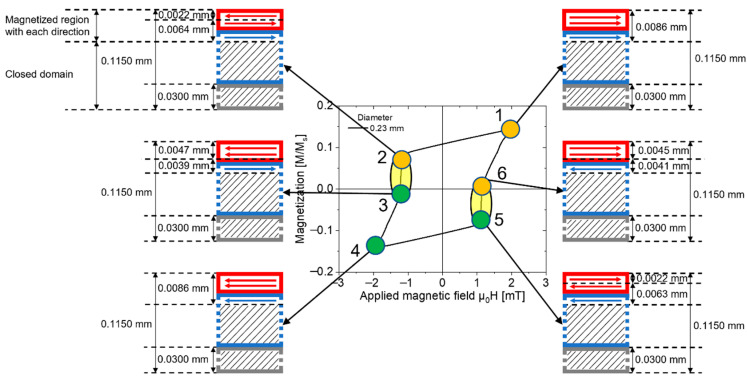
Magnetization process of 0.23 mm diameter Wiegand wire under an applied magnetic field of 2 mT.

The magnetization process and complex magnetic structure of the 0.23 mm diameter Wiegand wire under an applied magnetic field of 2 mT are shown in Figure 7. The magnetic structure of the Wiegand wire is expressed as a semi-cross-sectional structure in the axial direction, and its thickness is 0.1150 mm. The magnetized region with each direction of the Wiegand wire is indicated by a magnetic moment pointing to the left or right, and the closed domain is indicated by a set of oblique lines.

The numbers 1-6 are six points on the minor hysteresis loop. At Point 1, the thickness of the magnetized region with the right direction of the Wiegand wire was 0.0086 mm. Point 4 is symmetric to Point 1. Hence, the thickness of the magnetized region with the left direction at Point 4 was also 0.0086 mm. At Point 2, which is before the large Barkhausen jump, the surface of the Wiegand wire, whose coercivity is low, reverses during demagnetization. The thickness of the reversed volume was 0.0022 mm, and that of the unreversed volume was 0.0064 mm. At Point 3, which is after the large Barkhausen jump, the thickness of the reversed volume increased to 0.0047 mm, and that of the unreversed volume was 0.0039 mm. Point 5 and Point 6 are symmetric to Point 2 and Point 3, respectively. Thus, the magnetization process is similar, whereas the magnetization direction is the opposite.

The magnetization process in the 0.23-mm-diameter Wiegand wire under an applied magnetic field of 2 mT was analyzed using the semi-cross-sectional structure shown in Figure 7. In the initial state, the magnetic moments were distributed in arbitrary directions, and the Wiegand wire did not exhibit macroscopic magnetism. When a positive magnetic field of 2 mT is applied to the Wiegand wire, the thickness of the magnetized region with the right direction of the Wiegand wire is 0.0086 mm; that is, the direction of the magnetic moment is to the right, as shown in Figure 7 at Point 1. Gradually, the applied magnetic field was reduced until it dropped to zero. At this instant, the magnetic moments varied by reversible magnetization can be reverted back to their initial states, whereas the magnetic moments varied by irreversible magnetization cannot be reverted back to their initial states. The vector sum of all magnetic moments in the Wiegand wire is not zero; therefore, the magnetization is not zero. That is, when the applied magnetic field decreases to zero, residual magnetization occurs in the Wiegand wire.

Subsequently, the magnetic field was decreased in the opposite direction. The soft layer with low coercivity on the surface of the Wiegand wire first undergoes magnetization reversal. Consequently, the magnetization of the Wiegand wire continued to decrease. When the magnetic field was decreased to Point 2, the soft layer with a thickness of 0.0022 mm on the surface of the Wiegand wire was reversed. At this instant, the intensity of the magnetic field reached the critical value of the switching field of the Wiegand wire; thus, the Wiegand effect can occur. Then, the Wiegand wire can generate a large Barkhausen jump [9], and the magnetization state of the Wiegand wire varies abruptly from Point 2 to Point 3. A soft layer with a thickness of 0.0025 mm undergoes magnetization reversal during this process.

When the magnetic field decreased to −2 mT, that is, at Point 4, the 0.0086 mm thick region of the Wiegand wire is reversely magnetized, which is symmetrical to the state of Point 1 with respect to the origin. Gradually, the magnetic field is increased to zero; continue to apply the positive magnetic field until it reaches 2 mT. The magnetization state of the Wiegand wire transitions following the sequence Point 4–5–6–1, which is symmetrical to Point 1–2–3–4 with respect to the origin. At this instant, the periodic magnetization process of the Wiegand wire is completed under an applied magnetic field of 2 mT. The magnetization process is the same for all Wiegand wires with different diameters under different applied magnetic fields. Only the volumes of the regions of magnetization in the soft, middle, and hard layers vary in different states. Consequently, the magnetization process was not repeated.

## 5. Conclusions

In this study, the major and minor hysteresis loops of the five Wiegand wires with different diameters were measured using VSM. A simple magnetic structure for the Wiegand wire was proposed for the first time, and the dimensions of the soft, middle, and hard layers were defined. The diameter of the hard layer is 0.06 mm, and the thicknesses of the soft and middle layers decrease with decreasing diameter. However, the coercivity of the soft layer increases as the wire diameter decreases. For the first time, the thickness of the region with each direction of magnetization was determined by theoretical calculations, whereas previous studies were only qualitative. Under alternating magnetic fields of 2 to 15 mT in strength, the thicknesses of regions of magnetization with and without reversal during the magnetization process can be reliably calculated. The dynamic complex magnetic structure of the Wiegand wire and the different states of magnetization can be obtained from the theoretical model we developed, enabling researchers to develop applications for Wiegand wires. According to the conclusions, it can provide suggestions for producing the material of Wiegand wire. In the case of constant external conditions, it provides for the manufacture of Wiegand wires capable of producing a larger height of large Barkhausen jump. In other words, it can provide suggestions for producing a larger pulse voltage of the Wiegand wire.

## Figures and Tables

**Table 1:** The thicknesses of Δd, Δd1, and Δd2 of Wiegand wires with varying diameters under an applied magnetic field of 2 mT.

Magnetic Field (mT)	Points	D = 0.23 mm			D = 0.18 mm			D = 0.14 mm		
		Δd (mm)	Δd1 (mm)	Δd2 (mm)	Δd (mm)	Δd1 (mm)	Δd2 (mm)	Δd (mm)	Δd1 (mm)	Δd2 (mm)
2	1	0.0086	0.0000	0.0086	0.0070	0.0000	0.0070	0.0029	0.0000	0.0029
	2	0.0086	0.0022	0.0064	0.0070	0.0020	0.0050	0.0029	0.0019	0.0010
	3	0.0086	0.0047	0.0039	0.0070	0.0044	0.0026	0.0029	0.0024	0.0005
	4	0.0085	0.0000	0.0085	0.0070	0.0000	0.0070	0.0029	0.0000	0.0029
	5	0.0085	0.0022	0.0063	0.0070	0.0019	0.0051	0.0029	0.0019	0.0010
	6	0.0085	0.0045	0.0041	0.0070	0.0044	0.0026	0.0029	0.0024	0.0005

## Data Availability

Not applicable.
